# Hypospadias Risk from Maternal Residential Exposure to Heavy Metal Hazardous Air Pollutants

**DOI:** 10.3390/ijerph16060930

**Published:** 2019-03-15

**Authors:** Jeffrey T. White, Erin Kovar, Tiffany M. Chambers, Kunj R. Sheth, Erin C. Peckham-Gregory, Marisol O’Neill, Peter H. Langlois, Carolina J. Jorgez, Philip J. Lupo, Abhishek Seth

**Affiliations:** 1 Pediatric Urology, Norton Children’s Hospital, Louisville, KY 40207, USA; jeffrey.white@nortonhealthcare.org; 2 Department of Urology, University of Louisville School of Medicine, Louisville, KY 40202, USA; 3 Section of Hematology-Oncology, Department of Pediatrics, Baylor College of Medicine, Houston, TX 77030, USA; emkovar@bcm.edu (E.K.); Tiffany.Chambers@bcm.edu (T.M.C.); Erin.Peckham@bcm.edu (E.C.P.-G.); 4 Scott Department of Urology, Baylor College of Medicine, Houston, TX 77030, USA; Kunj.Sheth@bcm.edu (K.R.S.); cj129804@bcm.edu (C.J.J.); 5 Division of Pediatric Urology, Department of Surgery, Texas Children’s Hospital, Houston, TX 77030, USA; 6 Department of Pediatrics, Texas Children’s Hospital, Houston, TX 77030, USA; 7 Department Molecular and Cellular Biology, Baylor College of Medicine, Houston, TX 77030, USA; Marisol.oneill@ucsf.edu; 8 Birth Defects Epidemiology and Surveillance Branch, Texas Department of State Health Services, Austin, TX 78751, USA; peter.langlois@dshs.texas.gov

**Keywords:** congenital malformation, penis development, genitalia, pollutant, teratogen, hypospadias

## Abstract

*Objective*: Investigate whether residential prenatal exposure to heavy metal hazardous air pollutants (HMHAPs) is associated with an increased risk of hypospadias. *Methods:* Data on non-syndromic hypospadias cases (*n* = 8981) and control patients delivered in Texas were obtained from the Texas Birth Defects Registry and matched 1:10 by birth year. Average exposure concentrations of HMHAPs were obtained from the 2005 U.S. Environmental Protection Agency National-Scale Air Toxics Assessment and categorized into quintiles. Odds ratios and 95% confidence intervals were estimated. STROBE reporting guidelines were followed. *Results*: We observed associations between hypospadias and prenatal HMHAP exposure. Manganese demonstrated significant increased risk of hypospadias at the medium, medium-high and high exposure quintiles; lead in the medium-high and high exposure quintiles. Cadmium, mercury and nickel demonstrated a significant inverted “U-shaped” association for exposures with significant associations in the medium and medium-high quintiles but not in the medium-low and high quintiles. Arsenic and chromium demonstrated a significant bivalent association for risk of hypospadias in a lower quintile as well as a higher quintile with non-significant intermediate quintiles. *Conclusions*: Using data from one of the world’s largest active surveillance birth defects registries, we identified significant associations between hypospadias and HMHAP exposures. These results should be used in counseling for maternal demographic risk factors as well as avoidance of heavy metals and their sources.

## 1. Introduction

The most common external male genital malformation is hypospadias, affecting 1/125 to 1/300 live male births [1]. Hypospadias is defined as the abnormal closure of the genital folds during gestational weeks 8–14; it results in a urethral meatus on the ventral surface of the genital tubercle. Reports have demonstrated that alteration of the androgen signaling axis as well as the mesenchyme-to-epithelial transition (MET) of the genital tubercle will result in a hypospadias phenotype [2,3].

As the birth prevalence of hypospadias increases across the world [4,5,6,7,8,9,10], research into the association between pollutant exposure and hypospadias has defined a critical exposure period as the early first trimester of pregnancy [11]. Pollutants which interfere with MET during genitourinary development or hormonal cascades may play a role in hypospadias etiology. Endocrine-disrupting compounds such as Bisphenol A, atrazine, organic solvents and pesticides have been shown to be associated with hypospadias [1,12,13,14,15,16,17,18,19,20,21,22,23,24,25,26,27,28,29,30]. To our knowledge, however, the association between residential maternal exposure to heavy metal hazardous air pollutants (HAPs) with risk for hypospadias in a subsequently delivered fetus has not been studied.

HAPs, as defined by the Clean Air Act Amendments of 1990, are compounds associated with adverse health outcomes such as cancer and congenital anomalies [31]. The United States Environmental Protection Agency developed a nationwide database with modeled annual average concentrations of HAPs, termed the National-Scale Air Toxics Assessment (NATA) [32]. NATA is a national-level risk assessment based on the emissions of air toxics that produces census-tract level estimates of ambient and exposure concentrations for 180 air toxics, plus diesel particulate matter, which EPA assessed for noncancerous effects only. Using the concentration estimates for the 180 air toxics plus diesel particulate matter, NATA estimates cancer and non-cancer hazard risk for 138 HAPs.

By combining NATA data with the Texas Birth Defects Registry, we assessed the spatiotemporal associations of heavy metal hazardous air pollutants (HMHAPs) and hypospadias. We investigated whether prenatal exposure to HMHAPs is associated with an increased risk of hypospadias in a subsequently delivered fetus.

## 2. Materials and Methods

### 2.1. Study Population

Prior to receiving data, the study protocol was reviewed and approved by the Texas Department of State Health Services, and Baylor College of Medicine Institutional Review Boards. Hypospadias cases delivered in Texas between 1 January 1999 and 31 December 2008 were identified using the Texas Birth Defects Registry (TBDR) [33]. The TBDR, maintained by the Birth Defects Epidemiology and Surveillance Branch of the Texas Department of State Health Services, uses an active surveillance system to identify infants and pregnancies with birth defects within 1 year after delivery. Cases are identified through routine visits by TBDR staff members to all maternity hospitals, pediatric hospitals, birthing centers and midwife facilities in Texas. During these visits, discharge lists and unit logs are checked for parameters such as preterm births, stillbirths, ICD codes for birth defects or text descriptions relevant to birth defects. All diagnosed cases are coded using the Centers for Disease Control and Prevention modification of the British Pediatric Association Classification of Diseases and the World Health Organization’s International Classifications of Diseases, 9th Revision, Clinical Modification. These six-digit codes are commonly referred to as BPA codes. Infants with hypospadias have one of the following BPA codes: 752.600–752.607, 752.620, and 752.625–752.627.

Only non-syndromic, isolated hypospadias cases were included in our analyses to avoid heterogeneity [34]. Controls were unaffected live births who were born between 1 January 1999 and 31 December 2008. This group was randomly selected from Texas birth records and frequency matched by year of birth using a 10:1 ratio of controls to hypospadias cases. Control and case patients were excluded if data were missing (Supplementary Table S1). Overall, 8981 cases, and 89,810 controls were included in the study. 

### 2.2. Environmental Exposure Assessment

Estimated concentrations of HMHAPs were obtained from the 2005 NATA Hazardous Air Pollutant Exposure Model, version 5 (HAPEM5) [35]. This exposure model generates weighted-average exposure concentrations (μg/m^3^) for all nationwide census tracts, which are small subdivisions of a county created to provide a relatively uniform set of statistical demographic data. Census tract data maps are curated by the US Census Bureau and available at the following link: https://www.census.gov/geo/maps-data/maps/datamapper.html. As a result, HAPEM5 can track representatives of specified demographic groups as they move among indoor and outdoor microenvironments. This particular model was selected as it was the most recent assessment available for this population [36]. The census tract was based on maternal residence during time of delivery. To approximate exposure in the general population, HMHAP exposure levels (µg/m^3^) were categorized as quintiles based on the distribution in controls (low, low-medium, medium, medium-high, and high), as has been done in previous assessments of HAPs and adverse perinatal outcomes [37,38].

### 2.3. Potential Confounders

Information on the following potential confounders was obtained or calculated from birth or fetal death certificates provided from the Texas Department of State Health Services: plurality (1, 2, or ≥3 fetuses per pregnancy), maternal age (≤25 years, 26–30 years, 31–35 years, or >35 years), number of previous live births (0, 1, 2–3, or >3), maternal race/ethnicity (non-Hispanic White, non-Hispanic black, Hispanic, or other), birth weight (≤2500 g, 2501–3999 g, or >3999 g), maternal education (<12, 12, >12 years), maternal diabetes (no or yes), and maternal smoking (no or yes). 

Gestational age (≤37, 38–42, >42 weeks) was estimated by calculating the difference in weeks between the mother’s last menstrual period and date of delivery [39]. As the exposure assessment for HAPs was based on census tract-level estimates, a census tract-level estimate of percentage below poverty level was obtained from the U.S. Census 2005 Summary File [40]. These data were categorized into quartiles (low, medium-low, medium-high, and high) according to the percentage of census-tract control population below the poverty level.

### 2.4. Statistical Analysis

Logistic regression analyses were used to calculate the odds ratio (OR) and 95% confidence interval (CI) between hypospadias and each increasing maternal exposure category of selected HMHAPs, as well as selected covariates. Maternal race/ethnicity was chosen as a confounder in the final model based on the epidemiology of hypospadias, previous assessments of these exposures and related outcomes [41,42,43]. Birth year was included as a covariate since it was used to match cases and controls. *p*-values for trend were then calculated for the adjusted logistic regression models in order to assess relative differences between increasing exposure of the specific HMHAPs and hypospadias prevalence [42,43,44,45,46]. Associations between each HMHAP exposure quintile and non-syndromic isolated hypospadias cases were considered significant when *p* < 0.05. A sensitivity analysis was run excluding cases and/or controls from multiple-birth pregnancies: no effect was seen (data not shown). Patients that were excluded from individual analyses due to missing data for one of the confounding variables or HMHAP variables are included in Supplemental Table S1.

A weighted risk model was developed to assess the cumulative effect of HMHAPs with hypospadias. Having been used previously in genetic epidemiology studies, weighted risk scores account for the differences in each HMHAP’s effect size within the study population [47]. Quintile exposure of each metal was multiplied by the OR between hypospadias and each metal quintile and then summed over the seven HMHAPs to develop a cumulative exposure risk. Logistic regression was then performed to develop an OR and CI for increasing cumulative HMHAP exposure. *p*-values for trend were also calculated for this model. 

All analyses were carried out using Stata version 14.0 (Stata Corp., College Station, TX, USA). 

### 2.5. Reporting Guidelines

STrengthening the Reporting of OBservational studies in Epidemiology (STROBE) guidelines for case control studies were followed [48].

## 3. Results

There were 8981 cases of non-syndromic, isolated hypospadias delivered in Texas between 1 January 1999 and 31 December 2008. By matching birth year for hypospadias patients to live births without birth defects in Texas, 89,910 controls were randomly selected from birth certificate data. The demographics of these patients are presented in Table 1. Differences in demographic characteristics between control patients and hypospadias cases were noted. There were statistically significant associations between risk for hypospadias and plurality, maternal age, and maternal education. A statistically significant inverse association was seen between risk for hypospadias and number of live births, gestational age, birth weight and census tract poverty density. Race/ethnicity was significantly associated with hypospadias; birth prevalence was highest in non-Hispanic whites. Maternal diabetes or smoking was associated with a statistically significantly increased risk for hypospadias.

Estimated levels of HMHAPs were categorized based on levels in controls at the <20th, 20th to 40th, 40th to 60th, 60th to 80th and >80th centiles and were respectively labeled as low, medium-low, medium, medium-high and high exposure groups. Notably, all evaluated HMHAPs showed a significant association with hypospadias risk after adjusting for potential confounders (Table 2). Additionally, there was a significant trend between increasing exposure and hypospadias prevalence for arsenic, chromium, lead, manganese and mercury. However, the exposure–disease risk relationship did differ by specific pollutant. For example, lead was significantly associated with hypospadias risk only in the medium-high and high exposure quintiles and manganese and mercury for medium, medium-high and high exposure quintiles. Cadmium and nickel demonstrated an inverted “U-shaped” exposure–disease risk relationship, where significant associations were seen in the medium and medium-high exposure groups. Arsenic and chromium demonstrated a significant bivalent association for increased risk of hypospadias: arsenic for the medium and high quintiles versus chromium for the medium-low, medium-high and high quintiles. 

To assess the cumulative effect of HMHAPs with hypospadias, a weighted risk model was developed. Quintile exposure of each metal was multiplied by the OR for each metal and then summed over the seven HMHAPs to develop a cumulative exposure risk. Logistic regression was then performed to develop an OR and CI for increasing cumulative HMHAP exposure (Table 3). This analysis demonstrated significant associations with increasing exposure in the medium, medium-high and high quintiles (OR = 1.11, 95% CI: 1.03–1.19; OR = 1.14, 95% CI: 1.06–1.22; OR = 1.11, 95% CI: 1.03–1.20, respectively).

## 4. Discussion

This report examined the risk of hypospadias based on residential exposure to HMHAPs in Texas. Significant associations between HMHAPs and hypospadias were demonstrated with maximal risk of 1.2-fold. Additionally, combined exposure to all seven HMHAPs showed a significant 1.1-fold risk for hypospadias. Similar results have been seen in multiple prior reports regarding the association of residential environmental exposures with male genital anomalies [49,50]. Other studies which have looked at the risk of occupational exposure to heavy metals in developing hypospadias have shown stronger associations; however, the occupational setting confers much higher exposure levels than residential environmental sources [24,51,52]. Finally, hypospadias was not stratified by severity in order to reduce misclassification bias; however, as demonstrated by other reports, the magnitude of association between hypospadias and its risk factors progressively increases with hypospadias severity [24,53,54]. The authors accepted an underestimated effect to minimize bias in this study.

Three different patterns of hypospadias risk based on residential exposure levels were identified. First, several HMHAPs such as lead, manganese and mercury, demonstrated significant associations with increasing exposure. Manganese and mercury showed a small decrease in association at the highest exposure while lead was the only HMHAP with a 1.2-fold risk at the highest exposure. Second, an inverted “U-shaped” risk was observed for cadmium and nickel. Interestingly, this risk profile has been seen in prior studies correlating pollutant exposure with male genital malformations [49]. For these HMHAPs, the highest exposure groups may reflect a more severe (i.e., multiple or syndromic anomalies) or a lethal phenotype. All syndromic patients were excluded from this analysis; therefore, such a severe effect would not be observed. Alternatively, the weakened association with higher exposures may be due to misclassification or uncontrolled residual confounding despite adjustment for potential confounders, as seen in a prior study [55]. Third, bivalent risk was discovered for arsenic and chromium. The OR and CI differences for the non-significant middle exposure groups are very similar (e.g., Arsenic medium OR = 1.08, 95% CI: 1.01–1.16, versus Arsenic medium-high OR = 1.01, 95% CI: 0.94–1.09).

Each heavy metal demonstrated a significant association with hypospadias when exposure was categorized into quintiles. When HMHAP exposure was considered as a continuous variable, all heavy metals except cadmium and nickel demonstrated a significant association with hypospadias. To assess whether this hypospadias risk could be cumulative, hypospadias cases with multiple metal exposures were compared to control patients with multiple metal exposures. Each metal exposure was weighted by its corresponding OR to develop a continuous variable for weighted risk. This cumulative exposure model demonstrated significant risk for hypospadias with increasing exposure, with a small decrease in risk at the highest exposure. This small decrease could demonstrate the possibility that high exposures to multiple HMHAPs may cause a syndromic fetus or severe anomalies not compatible with life.

Each of these heavy metals has been implicated as a risk factor in human studies of various congenital anomalies. However, only arsenic, cadmium, lead, mercury and nickel have been evaluated in relation to hypospadias risk in previous assessments. One report from India demonstrated statistically significantly higher maternal as well as child blood levels of cadmium and lead for boys with hypospadias; however, the sample size was too small to calculate a measure of association [50]. A report from Australia described an adjusted OR (95% CI) of 2.59 (1.28–5.23) for risk of hypospadias due to maternal occupational exposure to heavy metals [24]. Unfortunately, these heavy metals were not identified but were based on inferred exposure from a prior published job–exposure matrix [51]. A study conducted in Sweden concluded that there was no association between maternal occupational exposure to nickel and hypospadias [56]. Notably, each of these studies was based on occupational exposures rather than residential-based environmental exposures (as evaluated in our assessment), which are characterized by higher exposure levels [52]. Thus, higher measures of association would be expected in studies of occupational exposures. A single report describes residential maternal chromium and cobalt exposure due to one mother’s hip arthroplasty implant elevating fetal chromium and cobalt levels [57]. In this case, the fetus was born with distal hypospadias; it is difficult to extrapolate such anecdotal data.

Weaknesses of the study include the retrospective design and the lack of mechanistic evaluations. The retrospective design of this study introduces several possible information biases. First, the use of residential information to estimate exposure rather than using a biomarker of exposure may introduce bias. However, there are few, if any, population-based resources to obtain relevant tissues to estimate exposure for hypospadias. Biomarkers for heavy metal exposures include markers of exposure (whole blood levels of the heavy metal), pathologic markers of toxicity (blood or urine levels of altered physiology) or markers of susceptibility (polymorphisms or copy number variants of enzymes which metabolize or eliminate a heavy metal from the body). These investigations are costly and not feasible on a population level. Therefore, using residential information is an important first step in ascertaining these exposure–disease risk relationships. Second, because data for maternal residence at conception were not available, maternal residence at delivery was used as a proxy. Thus, the potential for misclassification of exposure levels exists resulting in an altered estimation of effects. However, it has been shown that residential mobility during pregnancy is not expected to result in biased exposure assessment of HAPs in this population [58]. Third, geographical correlation of cases and controls with exposure data is reliant on accurate and true reporting of residential data. Within certain populations (e.g., undocumented individuals), residential data may be incorrect. These weaknesses, however, are inherent in all retrospective studies relying on residential data [11,49,55,58,59,60,61,62,63,64,65,66]. Finally, this report does not address the mechanism of action of the HMHAPs. Heavy metals have been reported to transfer through the placenta directly from mother to fetus and then exert an endocrine-disrupting potential to affect male birth anomalies such as hypospadias, cryptorchidism, and infertility [67,68,69,70]. The authors hope that future studies will be able to elucidate these mechanistic relationships. These weaknesses should not prevent extrapolation of these data to other populations.

The strengths of this study include the population-based study design with the large number of hypospadias cases identified by an active surveillance system. This data set included stillbirths and elective pregnancy terminations; other data sets which do not include these cases are more susceptible to selection bias. Most characteristics of our study population were similar to those described in prior studies: increased risk of hypospadias was seen with increasing maternal age [54,71], plurality [72,73], primiparity [54,74], maternal education level [49], low birth weight [54,72,73] and early term birth [54,75,76]. Additionally, maternal smoking and maternal diabetes increased risk of hypospadias. Both diabetes and smoking have been heavily debated with some studies showing increased risk and others showing reduced risk [49,54,72,74,75,76,77,78,79,80,81,82,83,84,85,86,87]. Non-Hispanic white patients demonstrated a higher risk for hypospadias while non-Hispanic blacks and Hispanics showed a reduced risk for hypospadias [30]. Interestingly, increasing poverty density showed a decreased risk of hypospadias. This result is likely due to a confounding socioeconomic disparity where geographical areas of higher poverty, such as city centers, demonstrate an over-representation of races which have lower risk of hypospadias. Prior reports lack clarity on this subject with some showing an association and others showing none [88,89,90].

## 5. Conclusions

To our knowledge, this is the first study to examine the association of individual and cumulative HMHAP residential maternal exposure with risk for hypospadias. HMHAPs are not well regulated or monitored, highlighting the need for better understanding of the effects of exposure to these pollutants. Considering our results, future studies are needed to test etiologic hypotheses regarding HMHAPs and hypospadias as well as develop biomarkers of exposure which can be tested in fertile women to aid in prevention and patient education of potential congenital anomalies of their offspring.

## Figures and Tables

**Table 1:** Demographics of hypospadias cases and control patients in Texas.

Demographic Characteristic	Controls	Cases	Odds Ratio (95% CI)	*p*
**Plurality**				
1	87,408	8553	1.00 (Ref)	
2	2304	409	1.81 (1.63–2.02)	<0.001 *
3+	94	19	2.07 (1.26–3.38)	0.004 *
**Maternal Age (years)**				
≤25	43,057	3861	1.00 (Ref)	
26–30	23,523	2456	1.16 (1.10–1.23)	<0.001 *
31–35	15,979	1791	1.25 (1.18–1.33)	<0.001 *
>35	7244	872	1.34 (1.24–1.45)	<0.001 *
**Previous Live Births**				
0	34,291	4099	1.00 (Ref)	
1	27,431	2580	0.79 (0.75–0.83)	<0.001 *
2–3	22,177	1856	0.70 (0.66–0.74)	<0.001 *
4+	3788	227	0.50 (0.44–0.57)	<0.001 *
**Race/ethnicity**				
Non-Hispanic white	32,624	4815	1.00 (Ref)	
Non-Hispanic black	10,076	1200	0.81 (0.75–0.86)	<0.001 *
Hispanic	43,403	2624	0.41 (0.39–0.43)	<0.001 *
Other	3565	328	0.62 (0.55–0.70)	<0.001 *
**Gestational Age (weeks)**				
Full term (38–42)	68,893	6391	1.00 (Ref)	
Early term (≤37)	16,880	2255	1.44 (1.37–1.52)	<0.001 *
Post-term (>42)	3734	301	0.87 (0.77–0.98)	0.022 *
**Birth Weight (g)**				
Normal (2501–3999)	75,403	6911	1.00 (Ref)	
Low (≤2500)	6222	1422	2.49 (2.34–2.65)	<0.001 *
High (>3999)	8138	645	0.86 (0.80–0.94)	0.001 *
**Education**				
Less than 12 years	27,701	1812	1.00 (Ref)	
12 years	26,116	2611	1.53 (1.44–1.63)	<0.001 *
Greater than 12 years	35,070	4476	1.95 (1.84–2.07)	<0.001 *
**Poverty density**				
Low	21,731	2827	1.00 (Ref)	
Medium-low	20,484	2092	0.79 (0.74–0.83)	<0.001 *
Medium-high	24,842	2350	0.73 (0.69–0.77)	<0.001 *
High	22,753	1712	0.58 (0.54–0.62)	<0.001 *
**Maternal diabetes**				
No	86,765	8636	1.00 (Ref)	
Yes	3045	345	1.14 (1.02–1.28)	0.025 *
**Maternal smoking**				
No	84,135	8359	1.00 (Ref)	
Yes	5280	576	1.10 (1.00–1.20)	0.039 *

Note: * denotes statistical significance.

**Table 2:** Unadjusted and Adjusted Odds Ratio (95% CIs) for associations between HMHAPs and hypospadias.

HMHAP Exposure	Pollutant Level (μg/m^3^)	Cases/Controls	Unadjusted OR (95% CI)	Adjusted OR (95% CI) ^a^	*p*-Trend ^b^
**Arsenic**					
Low	0.00013–0.00036	1708/16,778	1.00 (Ref)	1.00 (Ref)	
Medium-low	>0.00036–0.00042	1631/16,749	0.96 (0.89–1.03)	1.00 (0.93–1.07)	<0.001 *
Medium	>0.00042–0.00052	1747/16,798	1.02 (0.95–1.10)	1.08 (1.01–1.16)	
Medium-high	>0.00052–0.00068	1585/16,698	0.93 (0.87–1.00)	1.01 (0.94–1.09)	
High	>0.00068–0.0054	1787/16,810	1.04 (0.97–1.12)	1.18 (1.10–1.27)	
**Cadmium**					
Low	0.000037–0.000044	1506/16,761	1.00 (Ref)	1.00 (Ref)	
Medium-low	>0.000044–0.000054	1759/16,694	1.17 (1.09–1.26)	1.04 (0.96–1.11)	0.343
Medium	>0.000054–0.000067	1890/16,735	1.26 (1.17–1.35)	1.13 (1.05–1.21)	
Medium-high	>0.000067–0.000089	1821/16,887	1.20 (1.12–1.29)	1.11 (1.03–1.19)	
High	>0.000089–0.0049	1482/16,756	0.98 (0.91–1.06)	1.00 (0.93–1.08)	
**Chromium**					
Low	0.000041–0.00031	1632/16,734	1.00 (Ref)	1.00 (Ref)	
Medium-low	>0.00031–0.00044	1834/16,816	1.12 (1.04–1.20)	1.08 (1.00–1.16)	0.006 *
Medium	>0.00044–0.00061	1671/16,773	1.02 (0.95–1.10)	1.05 (0.97–1.12)	
Medium-high	>0.00061–0.00080	1722/16,730	1.06 (0.98–1.13)	1.12 (1.04–1.20)	
High	>0.00080–0.014	1599/16,780	0.98 (0.91–1.05)	1.10 (1.02–1.19)	
**Lead**					
Low	0.00049–0.0012	1599/16,725	1.00 (Ref)	1.00 (Ref)	
Medium-low	>0.0012–0.0015	1577/16,804	0.98 (0.91–1.06)	0.99 (0.92–1.07)	<0.001 *
Medium	>0.0015–0.0019	1684/16,748	1.05 (0.98–1.13)	1.04 (0.97–1.12)	
Medium-high	>0.0019–0.0025	1753/16,794	1.09 (1.02–1.17)	1.13 (1.05–1.21)	
High	>0.0025–0.034	1845/16,762	1.15 (1.07–1.24)	1.20 (1.11–1.28)	
**Manganese**					
Low	0.00058–0.00061	1491/16,783	1.00 (Ref)	1.00 (Ref)	
Medium-low	>0.00061–0.00075	1703/16,743	1.14 (1.06–1.23)	1.06 (0.98–1.14)	<0.001 *
Medium	>0.00075–0.00094	1796/16,759	1.21 (1.12–1.30)	1.12 (1.04–1.20)	
Medium-high	>0.00094–0.0012	1813/16,782	1.22 (1.13–1.31)	1.18 (1.10–1.27)	
High	>0.0012–0.022	1655/16,766	1.11 (1.03–1.20)	1.11 (1.03–1.20)	
**Mercury**					
Low	1.69 × 10^−9^–6.03 × 10^−6^	1585/16,787	1.00 (Ref)	1.00 (Ref)	
Medium-low	>6.03 × 10^−6^–0.000020	1693/16,775	1.07 (0.99–1.15)	1.06 (0.99–1.14)	0.006 *
Medium	>0.000020–0.000038	1895/16,745	1.20 (1.12–1.29)	1.16 (1.08–1.24)	
Medium-high	>0.000038–0.000073	1764/16,814	1.11 (1.03–1.19)	1.14 (1.06–1.23)	
High	>0.000073–0.0016	1521/16,712	0.96 (0.90–1.04)	1.08 (1.00–1.16)	
**Nickel**					
Low	0.000065–0.00022	1648/16,737	1.00 (Ref)	1.00 (Ref)	
Medium-low	>0.00022–0.00046	1650/16,791	1.00 (0.93–1.07)	1.03 (0.95–1.10)	0.071
Medium	>0.00046–0.00072	1838/16,759	1.11 (1.04–1.19)	1.14 (1.06–1.22)	
Medium-high	>0.00072–0.0011	1767/16,774	1.07 (1.00–1.15)	1.09 (1.02–1.17)	
High	>0.0011–0.029	1555/16,772	0.94 (0.88–1.01)	1.04 (0.97–1.12)	

^a^ Adjusted for maternal race/ethnicity and birth year; ^b^ For the adjusted model; * denotes statistical significance.

**Table 3:** Odds Ratios (95% CI) for associations between HMHAP-weighted risks and hypospadias.

HMHAP-Weighted Risk	OR (95% CI) ^a^	*p*-Trend ^b^
Low	1.00 (Ref)	
Medium-Low	1.05 (0.98–1.13)	<0.001 *
Medium	1.11 (1.03–1.19)	
Medium-High	1.14 (1.06–1.22)	
High	1.11 (1.03–1.20)	

^a^ Adjusted for maternal race/ethnicity and birth year; ^b^ For the adjusted model; * denotes statistical significance.

## Supplementary Materials

The following are available online at https://www.mdpi.com/1660-4601/16/6/930/s1, Table S1: Patients excluded due to missing data.
