# Supplementary material for: Hypospadias Risk from Maternal Residential Exposure to Heavy Metal Hazardous Air Pollutants
Source: Int J Environ Res Public Health. 2019 Mar 15;16(6):930. doi: 10.3390/ijerph16060930 (PMC6466330; doi:10.3390/ijerph16060930)
Supplement: Supplementary file 1 [file ijerph-16-00930-s001.pdf]

International Journal of
Environmental Research and Public Health

MDPI

Hypospadias Risk from Maternal Residential Exposure to Heavy Metal Hazardous Air Pollutants

Jeffrey T. White, Erin Kovar, Tiffany M. Chambers, Kunj R. Sheth, Erin C. Peckham-Gregory, Marisol O'Neill, Peter H. Langlois, Carolina J. Jorgez, Philip J. Lupo and Abhishek Seth

Table S1. Patients excluded due to missing data.

Demographics	Controls	Cases
Plurality	4	0
Maternal Age (years)	7	1
Previous Live Births	2,123	219
Race	142	14
Gestational Age (weeks)	303	34
Birth Weight (g)	47	3
Education	923	82
Poverty density	0	0
Maternal diabetes	0	0
Maternal smoking	395	46
**HMHAPs**		
Arsenic	5,977	523
Cadmium	5,977	523
Chromium	5,977	523
Lead	5,977	523
Manganese	5,977	523
Mercury	5,977	523
Nickel	5,977	523
